# Effects of Dietary Docosahexaenoic Acid Levels on the Growth, Body Composition, and Health of Liver and Intestine in Juvenile Tiger Puffer (*Takifugu rubripes*)

**DOI:** 10.3390/ani15111514

**Published:** 2025-05-22

**Authors:** Lu Zhang, Chenchen Bian, Ziling Song, Yang Liu, Jiahao Liu, Qiang Ma, Yuliang Wei, Mengqing Liang, Houguo Xu

**Affiliations:** 1 College of Fisheries and Life Sciences, Shanghai Ocean University, 999 Huchenghuan Road, Shanghai 201306, China; 2 State Key Laboratory of Mariculture Biobreeding and Sustainable Goods, Yellow Sea Fisheries Research Institute, Chinese Academy of Fishery Sciences, 106 Nanjing Road, Qingdao 266071, China

**Keywords:** n-3 polyunsaturated fatty acid, marine fish, nutrient requirement, fatty acid deposition, aquafeed

## Abstract

Docosahexaenoic acid (DHA), a long-chain polyunsaturated fatty acid, plays a critical role in animal growth, inflammatory regulation, lipid metabolism, and neurological functions. However, the optimal dietary requirement of DHA for tiger puffer remains unknown. This study systematically investigated the effects of different dietary DHA levels on the growth performance, body composition, hematological parameters and tissue physiology of tiger puffer (average initial body weight 17.78 ± 1.92 g). Six experimental diets with graded DHA concentrations (0.09%, 0.57%, 1.35%, 1.61%, 2.28%, and 3.08% dry matter) were formulated. The feeding experiment was carried out in a seawater flow-through system for eight weeks, with each diet assigned to three replicate tanks. Based on the regression analysis of weight gain and specific growth rate, the maximum values were observed at the dietary DHA level of 1.75% and 1.88%, respectively. Appropriate DHA levels also significantly improved the muscle protein synthesis and lipid metabolism, and strengthened the intestinal morphology. Furthermore, a threshold for efficient DHA deposition in muscle was identified, beyond which excess DHA (3.08%) may be β-oxidized and therefore largely wasted. In conclusion, the optimal dietary DHA level for juvenile tiger puffer should be within the range of 1.75–1.88%.

## Simple Summary

In aquaculture, determining the optimal dietary requirements for essential nutrients like docosahexaenoic acid (DHA) is critical for fish health and sustainable feed development. This study investigated the effects of six graded DHA levels (0.09–3.08% of dry matter) on juvenile tiger puffer (*Takifugu rubripes*), a high-value marine species. A dietary DHA level of 1.75–1.88% maximized the growth performance, enhanced the muscle protein synthesis, and improved the intestinal morphology. However, excessive DHA (3.08%) tended to result in oxidative stress, inflammation, and tissue damage. The DHA deposition in muscle plateaued beyond the threshold, suggesting that the excess DHA was metabolized rather than stored. These findings provide practical guidance for the management of dietary DHA in tiger puffer farming.

## 1. Introduction

Fatty acids, particularly long-chain polyunsaturated fatty acids (LC-PUFA) such as docosahexaenoic acid (DHA, 22:6n-3), eicosapentaenoic acid (EPA, 20:5n-3), and arachidonic acid (ARA, 20:4n-6), are essential components of animal cell membranes. They can lower the phase transition temperature of membranes and enhance membrane fluidity, thus playing a crucial role in maintaining the normal physiological functions of biological membranes [1]. Among LC-PUFA, DHA has been shown to be particularly critical for fish development, growth, survival, stress resistance and disease resistance, as well as for the development and function of the nervous system [2,3,4]. Previous studies have shown that the dietary DHA requirements vary significantly among different fish species. The optimum dietary DHA level for juvenile Atlantic bluefin tuna (*Thunnus thynnus*) was approximately 3% [5], whereas that for juvenile red sea bream (*Pagrus major*) [6] and striped jack (*Pseudocaranx dentex*) [7] was 4.29–7.28% and 1.60–2.20%, respectively. The lack of DHA in the feed resulted in the cessation of growth in common cockle (*Cerastoderma edule*) [8]. However, it has also been shown that excess dietary DHA supplementation could induce liver injury in zebrafish or inhibit the growth of abalone (*Haliotis discus hannai*) [9]. Therefore, it is crucial that dietary DHA must be at appropriate levels to maintain the growth performance and health status of fish.

Investigation of optimum dietary DHA levels is also very important for determining a suitable level of fish oil (FO) replacement by alternative lipid sources. Because of the relative shortage of FO, searching for sustainable alternative lipid sources has become a critical task in the aquaculture industry. Terrestrial lipids including poultry oil, beef tallow, and lard, along with plant-derived oils such as soybean oil, rapeseed oil, and palm oil, have gained prominence as alternative lipid sources in aquaculture feeds owing to their cost-effectiveness and reliable availability [10,11,12]. These substitutes, however, present nutritional limitations characterized by insufficient n-3 long-chain polyunsaturated fatty acids (LC-PUFA) and imbalanced lipid profiles. Such deficiencies may induce physiological disturbances in farmed fish and compromise the nutritional quality of the filet through altered fatty acid composition [13]. Establishing species-specific DHA requirements becomes crucial for determining the permissible thresholds of fish oil replacement in formulated diets while maintaining fish health and product value.

Tiger puffer is a high-value marine aquaculture species in Asia. However, the research on the nutritional requirements for this species has lagged behind. Based on growth performance, the optimal nutrient requirements for tiger puffer (*Takifugu rubripes*) have been determined as follows: crude protein, 41% [14]; crude lipid, 11% [15]; tryptophan, 0.507% [16]; ratio of calcium to phosphorus, 0.50 [17]; vitamin C, 29 mg/kg [18]; and calcium, 0.10–0.20% [19]. However, regarding the optimal dietary DHA level, which remains undefined. This study aimed to investigate the effects of dietary DHA levels on growth performance, body composition, fatty acid composition, hematological parameters and tissue physiology of juvenile tiger puffer. Leveraging the detailed genomic information of the tiger puffer [20,21], hepatic gene expression analysis was also conducted, in order to identify the physiological response of tiger puffer to dietary DHA at the transcriptional level. The findings will be helpful to the precise nutrition of tiger puffer and the development of sustainable feeds.

## 2. Materials and Methods

### 2.1. Ethical Approval

All sampling protocols in this study, as well as all fish rearing practices, were reviewed and approved by the Animal Care and Use Committee of Yellow Sea Fisheries Research Institute.

### 2.2. Experimental Diets

Based on the protein (41%) [14] and lipid (11.5%) [15] requirements of tiger puffer, six iso-nitrogenous (appr. 49% crude protein), iso-lipidic (appr. 11.6% crude lipid), and iso-energetic (appr. 20.9 kJ/g energy) experimental diets were formulated (Table 1). DHA-enriched oil was added to the basal diet at concentrations of 0.031% (DHA0), 0.663% (DHA1), 1.295% (DHA2), 1.927% (DHA3), 2.559% (DHA4), and 3.191% (DHA5), respectively. The rapeseed oil level was adjusted to balance the DHA oil level. The actual DHA content in the experimental diets was 0.09%, 0.57%, 1.35%, 1.61%, 2.28%, and 3.08%, respectively. Meanwhile, EPA and ARA enriched oils were added at constant levels in the experimental diets (Table 1). The DHA/EPA ratio in the six diets was 0.034, 0.723, 1.418, 2.103, 2.802, and 3.482, respectively, and the total DHA + EPA content was 0.945%, 1.580%, 2.209%, 2.844%, 3.473%, and 4.108% (of dry matter), respectively. Pellets with 1.0 mm diameter were prepared. The pellets were dried in an oven at 55 °C for 12 h. At last, all diets were packaged and stored at −20 °C before used.

### 2.3. Feeding Procedure and Sampling

Juvenile tiger puffer (initial average weight: 17.78 ± 1.92 g) were acclimated for 2 weeks with commercial feed at the Langya Experimental Base of the Yellow Sea Fisheries Research Institute (Qingdao, China). After 24 h fasting, fish were randomly distributed into 18 polyethylene tanks (300 L, triplicates per group, 25 fish/tank) and fed twice daily (08:00 and 18:00) to satiation for 8 weeks. Residual feed and feces were removed daily via siphoning, with two-thirds water exchanged. The water environmental parameters during the experiment included: temperature, 27–31 °C; salinity, 28–30; dissolved oxygen, more than 8 mg/L; pH, 7.6–7.8; and light: dark time, 12 h: 12 h.

At trial termination, fish were fasted for 24 h before sampling. The final body weight and survival were recorded. Three fish per tank were anesthetized with eugenol (1:10,000, *v*/*v*) and stored at −20 °C for whole-body proximate composition analysis. Eight additional fish per tank were dissected to measure the hepatosomatic (HSI) and viscerosomatic (VSI) indices. For tissue sample collection in each experimental tank, eight fish were randomly selected for blood collection (0.5–0.75 mL per fish), with blood from 2–3 fish pooled into one tube to yield three tubes per tank. At the same time, four pieces (1 × 1 cm) of liver apex were excised from four randomly selected fish per tank. For muscle sampling, two pieces of dorsal muscle (3 × 1.5 cm) were collected from each of three fish per tank to obtain a total of six pieces of muscle per tank. Additionally, the mid-intestine tissue (2 cm) was collected from four randomly selected fish per tank to ensure comprehensive tissue sampling for subsequent proximate composition and fatty acid analyses. Blood samples were collected from the caudal vein adjacent to the anal fin was centrifuged (4000× *g*, 10 min) after storage for 4 h at 4 °C to obtain the serum sample. Dorsal muscle, liver apex, and midgut tissues were flash-frozen in liquid nitrogen and stored at −76 °C for RT-qPCR. Liver and intestinal tissues from one fish per tank were collected for histology as described previously [22].

### 2.4. Analysis of the Proximate Composition of Fish

The proximate composition analysis of experimental diets and the whole body, muscle, and liver was performed according to the standard methods of Association of Official Analytical Chemists (AOAC, 1995) [23]. Moisture, crude protein (Kjeldahl method, N × 6.25), lipids (Soxhlet extraction with petroleum ether for whole-body/feed; chloroform-methanol method for muscle/liver), and ash (550 °C incineration for 8 h) contents were analyzed accordingly.

### 2.5. Biochemical Parameters of Serum

Standardized diagnostic assay kits (Nanjing Jiancheng Bioengineering Institute) were employed to analyze pooled serum samples collected from per tank, with measurements including lipid metabolism parameters (total cholesterol (T-CHO), triglycerides (TG), high- and low-density lipoprotein cholesterol (HDL-C & LDL-C), total bile acid (TBA)) and oxidative stress markers (malondialdehyde (MDA)) according to manufacturer protocols.

### 2.6. Fatty Acid Composition

Lipids extracted via chloroform-methanol were methylated with BF3-KOH/methanol and analyzed using gas chromatography (GC2010 Pro, Shimadzu, Kyoto, Japan) equipped with a flame ionization detector and SH-RT−2560 capillary column. Fatty acid compositions were expressed as % total fatty acids (TFA), following established protocols. More details can be found in our previous publications [24].

#### Determination of Absolute DHA Content in Diets by Gas Chromatography

The estimated (calculated based on formulation) DHA contents in each experimental diet (0.031%, 0.663%, 1.295%, 1.927%, 2.559%, and 3.191%) were used to calculate the concentration of DHA in 1 mL n-hexane when extracted from 200 mg diet sample (based on a moisture content of 4.17% in the diet). The calculated (estimated) DHA concentration in n-hexane after extraction for the six diets was 0.06, 1.27, 2.48, 3.69, 4.90, and 6.12 mg/mL, respectively. According to this estimation, a standard DHA solution grade was prepared using n-hexane and methyl docosahexaenoate. The graded DHA concentration was 0, 1.3, 2.5, 3.8, 5, and 6 mg/mL. The internal standard, methyl nonadecanoate (19:0), was initially added at a concentration of 1 mg/mL, based on the molecular weight ratio of DHA to 19:0. The concentration of 19:0 was adjusted according to the peak area ratio of DHA to 19:0 obtained from gas chromatography (GC), to make sure the peak area of both DHA and 19:0 was suitable. Following the first GC analysis, the 19:0 concentration was further adjusted based on the peak area, and the standard curve was generated using the adjusted values.

For each sample, 200 mg of the experimental diet was processed and the lipid was accurately extracted into 1 mL n-hexane. The internal standard 19:0 was then added. The ratio of peak area of DHA (ADHA) to that of 19:0 (A19:0) was determined using GC. The DHA content in the sample was calculated using the standard curve obtained before.

The DHA concentration (CDHA) and C19:0 concentration (C19:0) were calculated using the following equations:

$$C_{DHA}=a\times A_{DHA}$$

$$C_{19:0}=b\;\times \;A_{19:0}$$

where

$C_{DHA}$: concentration of DHA

$C_{19:0}$: concentration of 19:0

$A_{DHA}$: peak area of DHA

$A_{19:0}$: peak area of 19:0

a: absolute correction factor for DHA, representing the concentration of DHA per unit of peak area

b: absolute correction factor for 19:0, representing the concentration of 19:0 per unit of peak area

The ratio of DHA to 19:0 concentration was calculated as:

$$\frac{C_{DHA}}{C_{19:0}}=\frac{a}{b}\times \frac{A_{DHA}}{A_{19:0}}$$

A standard curve was plotted with $\frac{A_{DHA}}{A_{19:0}}$ as the *x*-axis and $\frac{C_{DHA}}{C_{19:0}}$ as the *y*-axis, and a/b will be a constant.

### 2.7. Histological Structure

Tissue (liver and intestine) samples from one fish each tank were used for the histological analysis according to the methods mentioned in our previous publications [22]. The tissue samples were subjected to hematoxylin and eosin (H&E) staining according to conventional histopathological protocols. The slices made were investigated and photographed with Digital Slide Scanner (3DHISTECH Ltd., Budapest, Hungary). All slices were analyzed using ImageJ 1.53c (Wayne Rasband National institutes of Health, Bethesda, MD, USA) for statistical analysis.

### 2.8. RNA Extraction and RT-qPCR Analysis

Total RNA isolation was performed on hepatic, muscular, and intestinal tissue specimens (n = 6 per experimental group) employing RNAiso Plus reagent (TaKaRa Biotechnology (Dalian) Co., Ltd., Dalian, China). The RNA purity was assessed using a Colibri microvolume spectrophotometer (Titertek Berthold, Pforzheim, Germany), based on the A260/A280 ratio (1.8–2.0). Reverse transcription was conducted with the PrimeScript™ RT Reagent Kit with gDNA Eraser (TaKaRa, Dalian, China) as per the manufacturer’s instructions. Custom oligonucleotide primers targeting at both experimental genes and endogenous controls (rpl13 and rpl19) [25] were commercially produced (TsingKe Biological Technology Co., Ltd., Qingdao, China), with their sequences detailed in Table 2. Primer validation tests demonstrated that the amplification efficiencies ranged from 95% to 110% when assessed through serial dilution analyses, accompanied by strong linear correlations (R^2^ > 0.99). The RT-qPCR was conducted using SYBR^®^ Premix Ex Taq TM (TaKaRa Biotechnology (Dalian) Co., Ltd., Dalian, China) and a quantitative thermal cycler (Roche LightCycler 96, Basel, Switzerland). The reaction mixture consisted of 2 μL cDNA template, 10 μL SYBR^®^ Premix Ex Taq TM (2×), 0.8 μL forward primer (10 μM), 0.8 μL reverse primer (10 μM), and 6.4 μL sterilized water. The thermal cycling program was as follows: 95 °C for 30 s, followed by 40 cycles of “95 °C for 5 s, 57 °C for 30 s, and 72 °C for 30 s”. After the amplification phase, melting curve analysis was performed (from 65 °C to 97 °C, incrementing by 6.4 °C per min) to confirm the specificity of the products. Each sample was run in triplicate. Gene expression levels were calculated using the RT-qPCR method: 2^−ΔΔCt^ [26].

### 2.9. Calculation and Statistics

#### 2.9.1. Calculation

Weight gain (g) = final body weight-initial body weight. Weight gain ratio (%) = (final body weight-initial body weight)/initial body weight × 100. Specific growth rate = (ln(final weight)-ln(initial weight))/days of experiment × 100. Feed conversion ratio = (final weight-initial weight)/total feed intake. Survival = final fish number/initial fish number × 100. Hepatosomatic index = liver weight/body weight × 100. Viscerosomatic index = viscera weight/body weight × 100. Condition factor = body weight/body length^3^ × 100.

#### 2.9.2. Statistics

Statistical analyses were conducted using SPSS 27.0.1 (Armonk, NY, USA) with percentage data normalized through arcsine transformation. Parametric assumptions were validated through Levene’s variance homogeneity testing prior to implementing one-way ANOVA. Significant intergroup variations (*p* < 0.05) were identified using Tukey’s post hoc method, with experimental outcomes expressed as mean values (triplicate observations) ± SEM. Regression models were selected based primarily on statistical significance (*p* < 0.05), with coefficient of determination (*R*^2^) serving as a secondary criterion when comparing models of equivalent significance.

## 3. Results

### 3.1. Growth Performance, Somatic Indices, and Body Composition

With increasing dietary DHA contents, the weight gain of experimental fish initially increased and then decreased (Table 3). The final body weight and weight gain were the highest in the DHA3 group, while the lowest values were observed in the DHA0 group. The weight gain and specific growth rate were the highest in the DHA1 and DHA3 groups, whereas the DHA0 group showed the lowest values. However, no significant (*p* > 0.05) differences were observed among the groups in terms of feed conversion ratio, survival, VSI, HSI, and condition factor.

**Figure 1:**
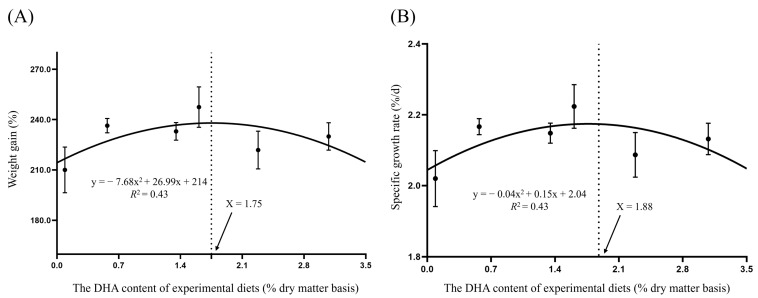
Regression analysis between dietary DHA level and weight gain (WG) (**A**) or specific growth rate (SGR) of (**B**) juvenile tiger puffer.

Based on the regression analysis of weight gain and specific growth rate (Figure 1), the maximum values were observed at dietary DHA levels of 1.75% and 1.88%, respectively.

The crude protein content in whole fish body was the highest in the DHA0 group, significantly (*p* ˂ 0.05) higher compared to the DHA4 group (Table 4). In terms of whole-body crude lipid content, the DHA3 group exhibited the highest value, which was significantly (*p* ˂ 0.05) higher compared to other groups. The whole-body crude lipid content in the DHA5 group was significantly (*p* ˂ 0.05) lower compared to DHA3, but was significantly (*p* ˂ 0.05) higher compared to DHA2. The DHA0, DHA1, and DHA4 groups had intermediate values.

In muscle, from DHA0 to DHA5, the total lipid content initially increased and then decreased. The highest and the lowest lipid content were observed in the DHA2 and DHA5 groups, respectively. Similarly, the crude protein content initially increased and then decreased, with increasing dietary DHA levels. The crude protein content in the DHA2 and DHA4 groups was the highest and significantly (*p* ˂ 0.05) higher compared to DHA0. No significant (*p* > 0.05) differences were observed in the moisture and ash contents among the groups. Furthermore, no significant (*p* > 0.05) differences were detected in crude protein, lipid, and moisture contents of the liver.

### 3.2. Serum Biochemical Parameters

The serum TG content was the highest in the DHA1 group, which was significantly (*p* ˂ 0.05) higher compared to other groups except DHA0 (Table 5). The TG content showed a decreasing trend from DHA2 to DHA5. From DHA0 to DHA4 groups, the MDA content showed an increasing trend. The MDA content was the highest in the DHA4 group, which was significantly (*p* ˂ 0.05) higher compared to DHA0. All groups showed no significant differences in the T-CHO, HDL-C, LDL-C and TBA contents.

### 3.3. Fatty Acid Profiles in the Whole Body, Muscle and Liver

In the whole body (Table 6) and liver (Table 7), from DHA0 to DHA5, the levels of 18:0, 22:5n-3, and DHA showed an increasing trend, whereas the levels of 20:0, 18:1n-9, 20:1n-9, 22:1n-9, 18:2n-6, 20:2n-6, ARA, and 18:3n-3 exhibited a decreasing trend.

In the muscle (Table 8), from the DHA0 to DHA5 groups, the levels of 16:0, 18:0, EPA, and DHA gradually increased, whereas the levels of 20:0, 18:1n-9, 20:1n-9, 22:1n-9, 20:2n-6, ARA, 18:3n-3, and 22:5n-3 gradually decreased.

### 3.4. Histological Structure of Tissues

**Figure 2:**
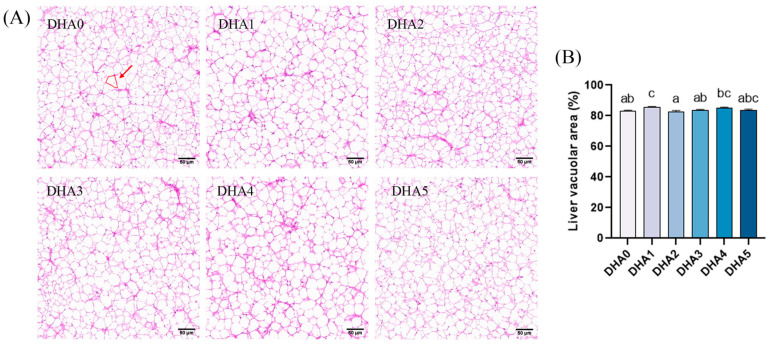
Effects of different levels of dietary DHA on liver histology of tiger puffer (*Takifugu rubripes*). (**A**) The H&E staining of liver transversal slices (20×); (**B**) the quantitative vacuolar area % in the liver of tiger puffer, with six view fields (20×) analyzed per slice. The area marked by the red circle represents the vacuolar area of a single vacuole. Data bars not sharing a same letter are significantly (*p* < 0.05) different.

The vacuolar area in the liver was the largest in the DHA1 group and the smallest in the DHA2 group, with no significant (*p* > 0.05) differences observed among the other groups (Figure 2).

**Figure 3:**
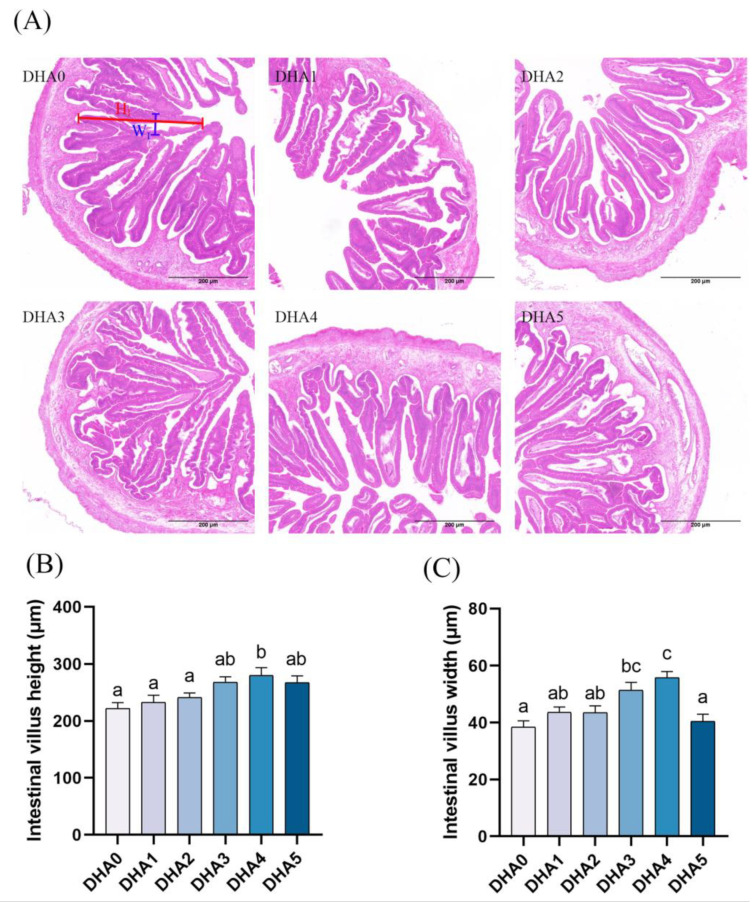
Effects of different levels of dietary DHA on the intestinal histology of tiger puffer (*Takifugu rubripes*). (**A**) the H&E staining of intestinal transversal slices (5×). (**B**,**C**) show the quantitative intestinal villus height and width, respectively, with six randomly selected intestine villus analyzed per slice; The blue and red lines show the height (HI) and width (WI) of intestine villus, respectively. Data bars not sharing a same letter are significantly (*p* < 0.05) different.

As dietary DHA content increased, the height of intestinal villi showed a dose-dependent increase, with the DHA4 group showing the highest villi height, followed by the DHA3 group (Figure 3). The width of intestinal villi showed a similar changing pattern in response to dietary DHA. However, when the dietary DHA content reached 3.08% (the DHA5 group), the villi width sharply decreased.

### 3.5. Gene Expression

#### 3.5.1. Liver Fibrosis and Inflammation Related Gene Expression

**Figure 4:**
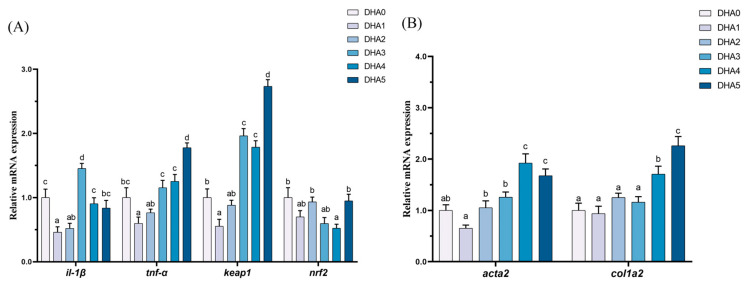
Effects of different levels of dietary DHA on the mRNA expression levels of inflammation (**A**) and liver fibrosis (**B**) related gene expression in the liver of tiger puffer (*Takifugu rubripes*). Data are presented as mean ± SEM (n = 3). According to Tukey’s test, bars not sharing the same letter are significantly (*p* < 0.05) different.

The expression levels of *il-1β* were the highest in the DHA3 group, followed by the DHA0 and DHA4 groups, with the DHA1 group showing the lowest levels (Figure 4). The *tnf-α* expression initially decreased and then increased with rising dietary DHA levels, peaking in the DHA5 group (the highest expression) and reaching the lowest level in the DHA1 group. The *keap1* expression had the same changing trend with *tnf-α*. The expression of *keap1* was highest in the DHA5 group and lowest in the DHA1 group. In contrast, the expression of *nrf2* was the lowest in the DHA4 group. The expression of *acta2* was the highest in the DHA4 and DHA5 groups, followed by the DHA2 and DHA3 groups, with the DHA1 group showing the lowest levels. The *col1a2* expression showed an increasing trend with increasing dietary DHA levels. The *col1a2* expression was the highest in the DHA5 group, followed by the DHA4 group.

#### 3.5.2. Intestinal Inflammation and Intestinal Barrier-Related Gene Expression

**Figure 5:**
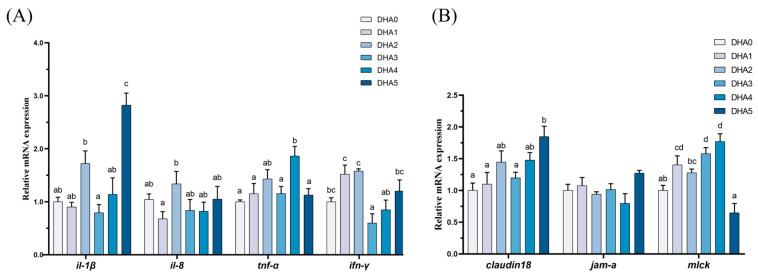
Effects of different levels of dietary DHA on the mRNA expression levels of intestinal inflammation-related (**A**) and intestinal barrier-related (**B**) gene expression in the intestine of tiger puffer (*Takifugu rubripes*). Data are presented as mean ± SEM (n = 3). According to Tukey’s test, bars not sharing the same letter are significantly (*p* < 0.05) different.

In the intestine, the expression levels of *claudin18*, and *mlck* showed an increasing trend in response to increasing dietary DHA levels (Figure 5). The expression of *il-1β* was the highest in the DHA5 group, followed by the DHA2 group, with the DHA3 group showing the lowest levels. For *il-8*, the DHA2 group showing the highest expression level, with the DHA1 group exhibiting the lowest, and the other four groups demonstrating intermediate values. The expression of *tnf-α* was the highest in the DHA4 group, followed by the DHA2 group, with the remaining four groups showing significantly lower levels (*p* < 0.05). The *ifn-γ* expression was the highest in the DHA1 and DHA2 groups, with the DHA3 group exhibiting the lowest levels, and the other three groups demonstrating intermediate values.

#### 3.5.3. Muscle Differentiation and Apoptosis Related Gene Expression

**Figure 6:**
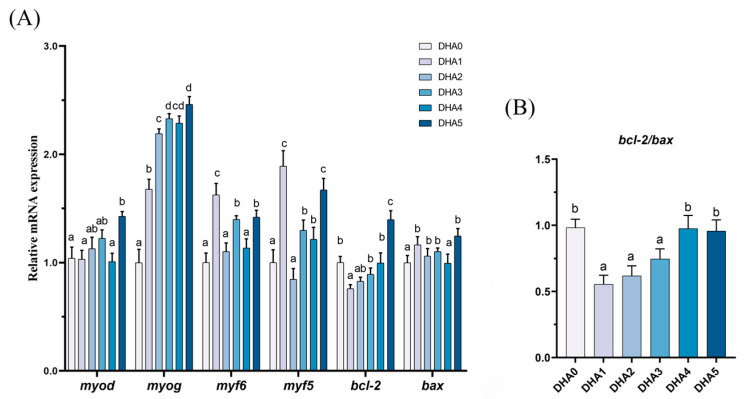
Effects of different levels of dietary DHA on the mRNA expression levels of cell differentiation and apoptosis related genes expression (**A**) and the *bcl-2/bax* ratio (**B**) in the muscle of tiger puffer (*Takifugu rubripes*). Data are presented as mean ± SEM (n = 3). According to Tukey’s test, bars not sharing the same letter are significantly (*p* < 0.05) different.

From DHA0 to DHA5, the expression of *myod* and *myog* exhibited an increasing trend in response to increasing dietary DHA levels, except for a slight decrease in the DHA4 group (Figure 6). For *myf6*, the DHA1 group exhibited the highest expression, followed by the DHA3 and DHA5 groups, with no significant differences among the DHA0, DHA2, and DHA4 groups. The expression of *myf5* was the highest in the DHA1 and DHA5 groups, followed by the DHA3 and DHA4 groups, with the lowest levels observed in the DHA0 and DHA2 groups. The *bcl-2*/*bax* ratio in the DHA1, DHA2, and DHA3 groups was significantly lower than that in the DHA0, DHA4, and DHA5 groups.

## 4. Discussion

DHA has been demonstrated to be an essential nutrient in several fish species, but the optimal dietary DHA level has been revealed to be different across species. Based on growth performance, the DHA requirement for juvenile blunt snout bream (*Megalobrama amblycephala*), Malabar grouper (*Epinephelus malabaricus*), and rainbow trout (*Oncorhynchus mykiss*) was 0.13–0.23% [27], 1.08% [28] and 2.07% [29], respectively. This was lower than the optimal dietary DHA content observed for juvenile tiger puffer in this study (1.75–1.88%). However, comparable DHA levels, 1.68–2.20% in diets of sobaity sea bream (*Sparidentex hasta*) led to a weight gain increase of 39.85–41.28% [30]. In Pacific white shrimp (*Litopenaeus vannamei*), the inclusion of 23.7 mg/kg (0.0024%) DHA in the diet resulted in a 10.2% increase in weight gain compared to the control group [31]. These results suggested that different species have distinct optimal DHA levels, and even minor deviations from these levels can significantly affect the growth performance. Furthermore, the previous studies also revealed that the effects of dietary DHA were dose-dependent. Both insufficient and excessive dietary DHA can adversely affect the fish growth. For example, replacing fish oil with soybean oil devoid of DHA significantly reduced the weight gain of cobia (*Rachycentron canadum*), and the growth performance was restored following the supplementation with DHA-rich algal meal [32]. Additionally, excessive dietary DHA has been shown to exert detrimental effects on the development across multiple fish species. A dietary DHA content exceeding 9.28% for Pacific cod (*Gadus macrocephalus*) larvae reduced the growth performance, likely due to peroxidation in larval tissues [33]. DHA in live foods promoted the development of brown sole (P*leuronectes herzensteini*) larvae but the survival was clearly depressed in larvae fed rotifer with high percentages of DHA (3.3%) [34]. A 5% increase in dietary DHA content in early-weaning diets dramatically increased the number of muscular lesions and the presence of ceroid pigment within hepatocytes of larval European sea bass (*Dicentrarchus labrax*) [35]. High dietary DHA contents also altered the oxidative status and caused muscular lesions in European sea bass larvae [36]. The adverse effect of excess dietary DHA was also observed in the present study. Therefore, precise management of dietary DHA content could be critical to not only the efficient use of DHA resource but also the animal performance.

Besides growth, the crude protein content in the muscle of tiger puffer was also increased by suitable dietary DHA levels, indicating a potential promotion of muscle development. In fish nutrition research, the promoting effect of DHA on muscle development has been well documented. Previous studies have shown that dietary DHA promoted the muscle fiber development in grass carp (*Ctenopharyngodon idella*) likely via the activation of MEK/ERK pathway [37]. Similarly, another study on blunt snout bream revealed that dietary DHA promoted the white muscle hyperplasia and muscle fiber development, which may be in association with the activation of AMPK/Sirt1 pathway [38]. In the present study, high dietary DHA levels up-regulated the mRNA expressions of *myod* and *myog*, indicating the hyperplasia and hypertrophy of myoblasts, respectively [39,40]. In addition, high dietary DHA levels led to an elevated *bcl-2*/*bax* ratio, indicating an anti-apoptotic status. This may further help to support the muscle tissue repair and regeneration, thereby preserving the muscle function integrity. However, it remains unclear whether the upregulation of muscle cell differentiation and apoptosis related genes expression in tiger puffer is directly mediated by the MEK/ERK pathway, which needs to be validated by future studies.

In response to increasing DHA levels, the total lipid content in the muscle first increased and then subsequently decreased. This non-linear response pattern has been consistently observed across multiple fish species, including silver pomfrets (*Pampus argenteus*) [41], blunt snout bream [27], and sobaity sea bream [30]. Although not significantly affected, the liver lipid content showed a similar changing pattern in response to dietary DHA. Similar results were also observed in Chinese tongue sole (*Cynoglossus semilaevis*) [42], yellowtail (*Seriola quinqueradiata*) [43], and Atlantic salmon (*Salmo salar*) [3]. The liver represents the primary lipid storage and metabolic organ in tiger puffer, with approximately 67% of hepatic tissue comprising adipocytes [44]. This unique lipid deposition mechanism, making the liver particularly sensitive to dietary DHA modulation and thus a critical target tissue for investigating lipid metabolism in this species [45]. The histological analysis of liver revealed that the vacuolar area in the liver of tiger puffer was minimized when the dietary DHA level reached 1.35%. Furthermore, increasing dietary DHA contents were associated with a reduction in serum triglyceride (TG) level. Similarly, Nile tilapia (*Oreochromis niloticus*) fed a DHA-enriched diet showed significant reductions in visceral somatic index, hepatic lipid content, and both serum and hepatic TG concentrations compared to those fed a control diet [46]. In gilthead sea bream (*Sparus aurata*) fed low-starch diets, dietary DHA supplementation lowered the total cholesterol levels [47]. In grass carp, DHA may induce lipolysis by activating the cAMP/PKA signaling pathway through endoplasmic reticulum (ER) stress [48]. Collectively, these findings highlight the lipid-lowering efficacy of an appropriate dietary DHA level in fish diets.

Regarding the fatty acid composition, as expected, the contents of DHA and EPA in the muscle increased with increasing dietary DHA levels, while the SFA content showed little change. Multiple studies have demonstrated that the SFA and MUFA in fish are primarily metabolized via β-oxidation to provide energy [49,50], while LC-PUFAs, such as DHA and EPA, are more likely retained for the formation of cell membranes and other physiological processes [51,52]. Similar results have been reported in silver sea perch [52], Atlantic Salmon [53] and Chinese tongue sole [54]. However, when dietary DHA level reached 3.08% in this study, the content of DHA and EPA in the muscle of experimental fish no longer increased, but the SFA content rose, suggesting that excess DHA may be β-oxidized prior to SFA. These results indicate the potential of using SFA and MUFA to spare LC-PUFA, which has been applied in some aquaculture practices.

To elucidate the physiological responses of tiger puffer to dietary DHA supplementation, hepatic gene expression studies were also performed. Specifically, high levels of dietary DHA inclusion significantly elevated the hepatic expression of pro-inflammatory cytokines (*il-1β* and *tnf-α*), suggesting an inflammatory effect. In addition, most groups exhibited upregulated expression of *keap1* and downregulated expression of *nrf2*. Under homeostasis, Keap1 sequesters Nrf2 in the cytoplasm, promoting proteasomal degradation to suppress antioxidant responses [55]. Oxidative stress (e.g., ROS) disrupts Keap1-Nrf2 binding, enabling Nrf2 nuclear translocation, heterodimerization with small MAF proteins, and activation of ARE-driven transcription for phase II enzymes, antioxidants, and transporters [56]. Therefore, the present results may indicate compromised endogenous antioxidant capacity and potential oxidative stress. This finding was further supported by the increases in serum MDA (a biomarker of lipid peroxidation) content depending on dietary DHA levels. Nevertheless, the DHA5 group presented an intriguing exception, showing a reduced MDA level despite a high DHA intake. Moreover, this group exhibited the unusual concurrent upregulation of *keap1* and *nrf2*. This paradoxical finding may be explained by the activation of mitochondrial quality control. Specifically, when the ROS generation surpasses cellular antioxidant capacity, the KEAP1/PGAM5 complex detects excessive mitochondrial superoxide/hydrogen peroxide production and initiates mitophagy to eliminate dysfunctional mitochondria [57]. Consequently, this selective removal of ROS-generating mitochondria would naturally result in decreased oxidative damage, thereby accounting for the observed reduction in serum MDA level.

Long-term inflammatory responses not only impair the function of tissue cells but also promote structural changes, including enhancement of hepatic fibrosis, a process characterized by the replacement of normal fibers with connective tissue [58]. In this study, as dietary DHA levels increased, key fibrotic genes *col1a2* and *acta2* in the liver showed consistently increasing trends, indicating potential hepatic fibrosis. The concurrent upregulation of pro-inflammatory cytokines (*il-1β* and *tnf-α*) and fibrosis marker genes (*col1a2* and *acta2*) suggest that dietary DHA may accelerate liver fibrosis progression by promoting inflammatory responses. Previous studies have demonstrated that *il-1β* can activate hepatic stellate cells through the NF-κB signaling pathway [59]. Meanwhile, *tnf-α* can significantly enhance the pro-fibrotic effect of *tgf-1β*, promoting extracellular matrix deposition [60]. The observed increase in *col1a2* expression in this study may be closely related to *tnf-α*-mediated activation of stellate cells. These results suggest that excessive dietary DHA may cause progressive liver damage through the inflammation-fibrosis axis, but the precise mechanisms need to be elucidated by further research.

The integrity of the intestinal barrier can prevent pathogenic bacteria, toxins, and large molecules from entering, thereby maintaining the internal homeostasis of the intestine. In this study, as the dietary DHA level increased, the height and width of intestinal villi significantly increased, indicating a positive effect of dietary DHA on intestinal morphology [61,62]. Previous studies have suggested that dietary DHA supplementation supported the functions of intestinal cells of European sea bass [63,64]. Similarly, in the present study, an appropriate level of DHA (1.61%) in the diet could protect the intestinal barrier function and alleviate the intestinal inflammation. However, excess DHA (primarily 3.08%) led to a reduction in villus height and width. This was consistent to the up-regulated gene expression of *claudin18* and *mlck*, which play important roles in disrupting endothelial barrier integrity and suppressing inflammation, respectively [65,66,67,68]), in this group. These results suggested that excess dietary DHA may cause damage to the intestinal barrier and induced an inflammatory response. To date, little information is available about the effects of dietary DHA on fish intestine, and future studies need to investigate the direct and indirect effects of DHA on the intestinal health.

## 5. Conclusions

In conclusion, appropriate levels of DHA (1.75–1.88%) can promote growth, enhance muscle development, and improve intestinal function in tiger puffer (*Takifugu rubripes*). However, excessive DHA can induce oxidative stress, inflammation, as well as damage of liver and intestinal structures.

## Figures and Tables

**Table 1:** Formulation and proximate composition of the experimental diets (% dry matter basis).

Ingredients	Dietary DHA Levels (% Dry Matter basis)					
	0.09	0.57	1.35	1.61	2.28	3.08
Fish meal	35.00	35.00	35.00	35.00	35.00	35.00
Poultry by-product meal	8.00	8.00	8.00	8.00	8.00	8.00
Soybean meal	14.00	14.00	14.00	14.00	14.00	14.00
Com gluten meal	10.00	10.00	10.00	10.00	10.00	10.00
Wheat meal	17.05	17.05	17.05	17.05	17.05	17.05
Brewer’s yeast	5.00	5.00	5.00	5.00	5.00	5.00
Mineral premix ^a^	0.50	0.50	0.50	0.50	0.50	0.50
Vitamin premix ^a^	1.00	1.00	1.00	1.00	1.00	1.00
Monocalcium phosphate	1.00	1.00	1.00	1.00	1.00	1.00
L-ascorbyl-2-polyphosphate	0.20	0.20	0.20	0.20	0.20	0.20
Choline chloride	0.20	0.20	0.20	0.20	0.20	0.20
Betaine	0.30	0.30	0.30	0.30	0.30	0.30
Ethoxyquin	0.02	0.02	0.02	0.02	0.02	0.02
Calcium propionic acid	0.10	0.10	0.10	0.10	0.10	0.10
Soya lecithin	1.00	1.00	1.00	1.00	1.00	1.00
DHA enriched oil ^b^	0.00	1.00	2.00	3.00	4.00	5.00
EPA enriched oil ^b^	1.20	1.19	1.17	1.16	1.14	1.13
ARA enriched oil ^b^	0.50	0.50	0.50	0.50	0.50	0.50
Rapeseed oil ^c^	4.93	3.94	2.96	1.97	0.99	0.00
Proximate composition						
Crude protein	49.10	49.10	49.10	49.10	49.10	49.10
Crude lipid	11.63	11.63	11.63	11.63	11.63	11.63
Ash	89.62	89.69	89.71	89.72	89.65	89.62
Energy KJ/g	21.35	21.16	20.95	20.94	21.33	21.07
DHA content	0.09	0.57	1.35	1.61	2.28	3.08

^a^ Mineral premix and vitamin premix, designed for marine fish, were purchased from Qingdao Master Biotech Co., Ltd. (Qingdao, China). ^b^ DHA (Purity, 63%), EPA (Purity, 64%) and ARA (Purity, 31%) enriched oil were purchased from Shaanxi GC Biotech Co, Ltd. (Xi’an, China). ^c^ Rapeseed oil was purchased from Shandong Luhua Group Co., Ltd. (Yantai, China).

**Table 2:** Sequences of the primers used in this study.

Primer	Sequence (5′−3′)	GenBank Reference	PL (bp)
*myod-F*	TTCATCATCACACCGAGGCG	NM_001032769.1	126
*myod-R*	GTCGGTCCACGTTTGTAGTCT		
*myog-F*	ACGCTAATCAGTGGGTCTGC	XM_003973605.3	89
*myog-R*	TAACTCGTGGCTTCGACAGG		
*myf6-F*	GATCTGCAAGCGCAAATCGG	NM_001032771.1	116
*myf6-R*	CCACGGTCTTCCTCTTGAGC		
*myf5-F*	GGAGTCCTCTGTCCAACTGC	NM_001032770.1	84
*myf5-R*	CGCTGCTGTAAACTGCGTTC		
*bax-F*	ACCGTTCCCAGTGCAAATCT	NC_042289.1	108
*bax-R*	TGGGAACACTTGAGCCCATC		
*bcl-2-F*	GGGCCGGATTATCGCTTTCT	NC_042306.1	111
*bcl-2-R*	TATTCCGTCATCCACTCCGC		
*acta2-6F*	ATTCCTCGTCCCTGTGTGGTC	XM_029845356.1	125
*acta2-6R*	GGCATCATCTCCAGCGAAGC		
*il-1β-F*	CATCACCCGCTGACCATGAA	NM_001280090.1	103
*il-1β-R*	CATCCCTGAACTCGGTGCTC		
*il-8-F*	CCTGCGGAGCCTCGGAGTG	AB125645.1	145
*il-8-R*	TGACATCTTCAGAGTGGCAATGATCTC		
*tnf-α-F*	CTACTGGAACGGAAGGCAAGAGATG	AB183465.1	100
*tnf-α-R*	GATGCGGCTCAGCGTGTAGTG		
*ifn-γ-F*	CTGTGATGACTCTTGGGGCT	XM_029825554.1	147
*ifn-γ-R*	TGTACCGCTGACAGGAGTTG		
*claudin18-F*	GACACAAGGGTCTGTGGCAG	AY554344.1	112
*claudin18-R*	ATGATCATCAGGGCTCGCAC		
*jam-a-F*	CAAAAACGGCGTGCCTCTAC	XM_003971244.3	122
*jam-a-R*	CCGAGTCCGACCTTGATGTT		
*mlck-F*	GACACGACTGGCACGCAGATC	XM_011621260.1	170
*mlck-R*	CAGATGACTCCGATGCTCCACATG		
*rpl19-F*	GTCTCATCATCCGCAAACC	XM_003964816	132
*rpl19-R*	TCTCAGGCATACGAGCATT		
*rpl13-F*	GTAACAGGTCCACAGAATCCC	XM_003969972	117
*rpl13-R*	CCTCAGTGCTGTCTCCCTTC		
*keap1-F*	ACCGTGATGGAGGAATCGAGC	XM_029851225.1	123
*keap1-R*	TCAGCTTACCAGAACCGAGGG		
*nrf2-F*	ACGCATTCGACAAACACGAC	XM_003961827.3	106
*nrf2-R*	CCGTACACAGACTTCCCAGG		
*col1a2-F*	TGTTGGAGAGGGTGGAAAGC	XM_011609483.2	136
*col1a2-R*	GACTCCCATTGGACCCTGAG		

*myod*: myogenic differentiation antigen; *myog*: myogenin; *myf6*: myogenic factor 6; *myf5*: myogenic factor 5; *bcl-2*: b-cell lymphoma-2; *bax*: bcl-2-associated x; *acta2*: actin alpha 2; *il-1β*: interleukin—1β; *il-8*: interleukin 8; *tnf-α*: tumor necrosis factor alpha; *ifn-γ*: interferon gamma; *jam-a*: junctional adhesion molecule a; *mlck*: myosin light chain kinase; *rpl19*: ribosomal protein l19; *rpl13*: ribosomal protein l13; *keap1*: Kelch-like ECH-associated protein 1; *nrf2*: nuclear factor erythroid 2; *col1a2*: collagen type I alpha 2 chain; PL: product length.

**Table 3:** Growth performance and somatic indices of experimental tiger puffer (*Takifugu rubripes*).

Parameters	DHA0	DHA1	DHA2	DHA3	DHA4	DHA5	Regression			
							Model	Equation	*R* ^2^	*p*
Initial weight (g)	17.69 ± 0.04	17.86 ± 0.09	17.77 ± 0.01	17.88 ± 0.09	17.77 ± 0.06	17.69 ± 0.03	/			
Final weight (g)	54.85 ± 1.35 ^a^	60.09 ± 0.29 ^bc^	59.16 ± 0.55 ^bc^	62.13 ± 1.3 ^c^	57.22 ± 2.02 ^ab^	57.19 ± 0.97 ^abc^	cubic	y = 0.24x^3^ − 3.17x^2^ + 12.49x + 45.34	0.52	0.018
Weight gain (g)	37.16 ± 1.36 ^a^	42.22 ± 0.31 ^bc^	41.39 ± 0.55 ^abc^	44.28 ± 1.27 ^c^	39.42 ± 1.03 ^ab^	40.67 ± 0.75 ^abc^	cubic	y = 0.24x^3^ − 3.13x^2^ + 12.29x + 27.80	0.50	0.017
Weight gain (%)	210.05 ± 7.86 ^a^	236.41 ± 2.48 ^b^	232.98 ± 3.03 ^ab^	247.46 ± 6.96 ^b^	222.02 ± 6.51 ^ab^	229.98 ± 4.71 ^ab^	quadratic	y = −7.68x^2^ + 26.99x + 214	0.43	0.029
Specific growth rate (%/d)	2.02 ± 0.05 ^a^	2.17 ± 0.01 ^b^	2.15 ± 0.02 ^ab^	2.23 ± 0.04 ^b^	2.09 ± 0.04 ^ab^	2.13 ± 0.03 ^ab^	quadratic	y = −0.04x^2^ + 0.15x + 2.04	0.43	0.027
Feed conversion ratio	1.40 ± 0.10	1.26 ± 0.07	1.29 ± 0.01	1.27 ± 0.01	1.25 ± 0.05	1.27 ± 0.08	cubic	y = −4.68 × 10^−3^x^3^ + 0.06x^2^ − 0.24x + 1.58	0.41	0.049
Survival (%)	96.00 ± 4.00	97.33 ± 4.62	98.67 ± 2.31	96.00 ± 4.00	98.67 ± 2.31	94.67 ± 6.11	/			
Viscerosomatic index (%)	12.72 ± 1.41	13.28 ± 0.65	12.64 ± 1.32	13.55 ± 1.82	13.12 ± 0.94	14.15 ± 1.68	/			
Hepatosomatic index (%)	8.54 ± 1.22	8.54 ± 1.22	8.00 ± 0.52	9.32 ± 1.77	8.30 ± 1.06	9.25 ± 1.61	/			
Condition factor (%)	3.14 ± 0.26	3.25 ± 0.27	3.23 ± 0.13	3.24 ± 0.22	3.42 ± 0.19	3.38 ± 0.21	/			

Data in the same row, not sharing the same superscript letter, are significantly different (*p* < 0.05). “/” indicates that no statistically significant model (*p* < 0.05) was obtained for this parameter.

**Table 4:** Effects of different levels of DHA on the proximate composition of whole body and fish tissues of juvenile tiger puffer (*Takifugu rubripes*).

Parameters	DHA0	DHA1	DHA2	DHA3	DHA4	DHA5	Regression			
							Model	Equation	*R* ^2^	*p*
**Whole body**										
Crude protein (% w.w.)	20.83 ± 0.63 ^b^	19.98 ± 0.33 ^ab^	19.78 ± 0.83 ^ab^	20.09 ± 1.16 ^ab^	18.95 ± 0.78 ^a^	19.54 ± 0.56 ^ab^	/			
Crude lipid (% w.w.)	8.22 ± 0.83 ^ab^	8.26 ± 0.67 ^ab^	7.57 ± 0.47 ^a^	9.93 ± 0.61 ^c^	7.94 ± 0.64 ^ab^	8.71 ± 0.68 ^b^	linear	y = 0.55x + 25.59	0.12	0.038
Moisture (%)	68.38 ± 0.92	69.26 ± 0.80	69.84 ± 1.14	68.81 ± 1.42	70.41 ± 0.50	69.50 ± 1.46	/			
Ash (% w.w.)	3.42 ± 0.17	3.13 ± 0.09	2.88 ± 0.21	2.93 ± 0.20	2.89 ± 0.45	2.90 ± 0.09	cubic	y = −0.01x^3^ + 0.15x^2^ − 0.71x + 4	0.50	0.017
**Muscle**										
Crude protein (% w.w.)	18.50 ± 0.37 ^a^	18.84 ± 0.47 ^ab^	19.51 ± 0.17 ^b^	19.35 ± 0.06 ^ab^	19.62 ± 0.40 ^b^	19.21 ± 0.14 ^ab^	quadratic	y = −0.09x^2^ + 0.79x + 17.75	0.63	<0.001
Total lipid (% w.w.)	1.06 ± 0.15 ^b^	1.03 ± 0.07 ^b^	1.44 ± 0.07 ^c^	1.11 ± 0.02 ^b^	1.00 ± 0.12 ^b^	0.76 ± 0.13 ^a^	cubic	y = 0.01x^3^ − 0.13x^2^ + 0.39x + 0.22	0.34	
Moisture (%)	79.19 ± 0.36	78.96 ± 0.32	78.46 ± 0.20	78.55 ± 0.19	78.12 ± 0.35	78.79 ± 0.19	cubic	y = 0.03x^3^ − 0.26x^2^ + 0.33x + 79.08	0.59	0.009
**Liver**										
Crude protein (% w.w.)	4.04 ± 0.09	3.97 ± 0.44	3.82 ± 0.30	3.81 ± 0.24	3.94 ± 0.48	3.90 ± 0.47	/			
Total lipid (% w.w.)	47.19 ± 3.20	48.34 ± 3.20	47.91 ± 3.28	48.76 ± 2.62	46.96 ± 3.87	44.18 ± 2.07	/			
Moisture (%)	30.55 ± 0.33	30.47 ± 0.41	30.35 ± 0.46	30.52 ± 0.26	30.36 ± 0.16	30.00 ± 0.47	/			

Data in the same row, not sharing a same superscript letter, are significantly different (*p* < 0.05). w.w.: wet weight. “/” indicates that no statistically significant model (*p* < 0.05) was obtained for this parameter.

**Table 5:** Effects of different levels of DHA on the serum biochemical parameters of juvenile tiger puffer (*Takifugu rubripes*).

Parameters	DHA0	DHA1	DHA2	DHA3	DHA4	DHA5	Regression			
							Model	Equation	R2	*p*
TG (mmol/L)	1.88 ± 0.18 ^ab^	2.36 ± 0.41 ^b^	1.50 ± 0.16 ^a^	1.39 ± 0.20 ^a^	1.49 ± 0.13 ^a^	1.10 ± 0.08 ^a^	linear	y = −0.18x + 2.2	0.30	< 0.001
T-CHO (mmol/L)	2.99 ± 0.19	3.49 ± 0.30	4.08 ± 0.22	3.78 ± 0.79	3.12 ± 0.18	3.28 ± 0.10	cubic	y = −0.04x^3^ + 0.44x^2^ − 1.27x + 1.73	0.44	< 0.001
HDL-C (mmol/L)	3.93 ± 0.55	3.84 ± 0.33	3.68 ± 0.44	3.87 ± 0.36	3.82 ± 0.50	3.71 ± 0.47	/			
LDL-C (mmol/L)	1.11 ± 0.16	1.05 ± 0.10	0.99 ± 0.15	1.01 ± 0.14	1.09 ± 0.18	1.00 ± 0.12	/			
TBA (μmol/L)	1.31 ± 0.15	1.15 ± 0.24	1.47 ± 0.16	1.31 ± 0.09	1.21 ± 0.13	1.60 ± 0.11	/			
MDA (nmol/mL)	24.84 ± 0.54 ^a^	28.32 ± 1.97 ^ab^	28.78 ± 1.58 ^ab^	28.79 ± 1.01 ^ab^	32.01 ± 1.15 ^b^	26.17 ± 1.78 ^ab^	/			

Data in the same row, not sharing a same superscript letter, are significantly different (*p* < 0.05). Abbreviations: TG: triglyceride; T-CHO: total cholesterol; HDL-C: high-density lipoprotein cholesterol; LDL-C: low-density lipoprotein cholesterol; TBA: total bile acid; MDA: malondialdehyde. “/” indicates that no statistically significant model (*p* < 0.05) was obtained for this parameter.

**Table 6:** Effects of different levels of dietary DHA on the fatty acid compositions in the whole body of juvenile tiger puffer (*Takifugu rubripes*) (% total fatty acids).

Fatty Acid	DHA0	DHA1	DHA2	DHA3	DHA4	DHA5	Regression			
							Model	Equation	*R* ^2^	*p*
14:0	1.21 ± 0.05	0.99 ± 0.07	1.06 ± 0.23	1.00 ± 0.03	1.07 ± 0.07	1.00 ± 0.20	/			
16:0	14.84 ± 0.23	14.73 ± 0.13	14.80 ± 0.67	14.78 ± 0.27	14.56 ± 0.42	14.25 ± 0.86	/			
18:0	4.66 ± 0.16 ^a^	5.53 ± 0.23 ^ab^	5.17 ± 0.24 ^ab^	5.31 ± 0.20 ^ab^	5.26 ± 0.23 ^ab^	6.24 ± 0.45 ^b^	cubic	y = 0.09x^3^ − 0.87x^2^ + 2.74x + 2.73	0.41	0.008
20:0	0.23 ± 0.00 ^b^	0.23 ± 0.01 ^b^	0.22 ± 0.01 ^ab^	0.18 ± 0.01 ^a^	0.19 ± 0.00 ^ab^	0.19 ± 0.02 ^ab^	cubic	y = 1.89 × 10^−3^x^3^ − 0.02x^2^ + 0.04x + 0.21	0.48	<0.001
SFA	20.94 ± 0.31	21.48 ± 0.37	22.64 ± 0.93	22.08 ± 0.37	21.68 ± 0.60	22.67 ± 1.41		/		
16:1n-7	0.15 ± 0.01	0.16 ± 0.01	0.15 ± 0.01	0.14 ± 0.01	0.15 ± 0.00	0.16 ± 0.01	cubic	y = 2.24 × 10^−3^x^3^ − 0.02x^2^ + 0.06x + 0.12	0.30	0.027
18:1n-9	31.36 ± 0.37 ^e^	27.61 ± 0.69 ^d^	22.92 ± 1.05 ^c^	21.06 ± 0.35 ^c^	16.98 ± 0.37 ^b^	12.01 ± 0.45 ^a^	linear	y = −3.57x + 34.73	0.95	<0.001
20:1n-9	1.33 ± 0.06 ^c^	1.29 ± 0.06 ^bc^	1.50 ± 0.16 ^c^	1.00 ± 0.03 ^ab^	1.02 ± 0.06 ^ab^	0.82 ± 0.05 ^a^	cubic	y = 9.57 × 10^−3^x^3^ − 0.12x^2^ + 0.36x + 1.07	0.60	<0.001
22:1n-9	0.26 ± 0.03 ^c^	0.23 ± 0.04 ^c^	0.28 ± 0.04 ^bc^	0.19 ± 0.00 ^ab^	0.19 ± 0.01 ^ab^	0.15 ± 0.01 ^a^	cubic	y = 1.84 × 10^−3^x^3^ − 0.02x^2^ + 0.05x + 0.23	0.52	<0.001
MUFA	33.10 ± 0.37 ^e^	29.29 ± 0.70 ^d^	24.84 ± 0.98 ^c^	21.96 ± 0.17 ^c^	18.22 ± 0.43 ^b^	13.13 ± 0.48 ^a^	linear	y = −3.7x + 36.68	0.95	<0.001
18:2n-6	15.61 ± 0.30 ^d^	13.37 ± 0.16 ^c^	11.28 ± 0.56 ^b^	11.03 ± 0.11 ^b^	9.36 ± 0.19 ^a^	7.88 ± 0.14 ^a^	cubic	y = −0.05x^3^ + 0.66x^2^ − 3.89x + 18.87	0.94	<0.001
20:2n-6	0.61 ± 0.03 ^ab^	0.69 ± 0.06 ^b^	0.69 ± 0.08 ^b^	0.50 ± 0.00 ^a^	0.52 ± 0.03 ^a^	0.46 ± 0.02 ^a^	cubic	y = 0.01x^3^ − 0.12x^2^ + 0.36x + 0.36	0.46	0.009
20:4n-6	1.85 ± 0.06 ^c^	1.78 ± 0.03 ^bc^	1.49 ± 0.09 ^a^	1.50 ± 0.04 ^a^	1.48 ± 0.05 ^a^	1.61 ± 0.08 ^ab^	cubic	y = 9.01 × 10^−3^x^3^ − 0.06x^2^ − 0.02x + 1.93	0.63	<0.001
n-6PUFA	18.07 ± 0.31 ^d^	15.84 ± 0.13 ^c^	13.46 ± 0.45 ^b^	13.03 ± 0.11 ^b^	11.36 ± 0.21 ^a^	9.95 ± 0.15 ^a^	cubic	y = −0.03x^3^ + 0.48x^2^ − 3.55x + 21.16	0.95	<0.001
18:3n-3	2.81 ± 0.03 ^f^	2.39 ± 0.11 ^e^	1.86 ± 0.11 ^d^	1.62 ± 0.01 ^c^	1.19 ± 0.03 ^b^	0.70 ± 0.03 ^a^	cubic	y = −0.01x^3^ + 0.13x^2^ − 0.79x + 3.49	0.99	<0.001
20:5n-3	5.20 ± 0.12	5.23 ± 0.22	5.05 ± 0.47	5.52 ± 0.08	5.57 ± 0.11	5.50 ± 0.13	/			
22:5n-3	3.11 ± 0.12 ^a^	3.59 ± 0.02 ^a^	3.92 ± 0.44 ^a^	4.85 ± 0.14 ^b^	5.02 ± 0.16 ^b^	5.74 ± 0.23 ^b^	cubic	y = −0.03x^3^ + 0.27x^2^ − 0.2x + 3.08	0.79	<0.001
22:6n-3	4.18 ± 0.14 ^a^	9.85 ± 0.11 ^b^	13.51 ± 1.22 ^c^	20.59 ± 0.20 ^d^	26.19 ± 0.94 ^e^	31.46 ± 1.05 ^f^	linear	y = 5.17x − 0.81	0.91	<0.001
n-3PUFA	15.30 ± 0.22 ^a^	21.06 ± 0.23 ^b^	26.52 ± 0.42 ^c^	32.57 ± 0.33 ^d^	37.97 ± 1.15 ^e^	43.99 ± 0.50 ^f^	cubic	y = −0.27x^3^ + 2.68x^2^ − 2.15x + 15.32	0.87	<0.001
DHA/EPA	0.80 ± 0.03 ^a^	1.89 ± 0.05 ^b^	2.67 ± 0.04 ^c^	3.74 ± 0.08 ^d^	4.70 ± 0.15 ^e^	5.97 ± 0.10 ^f^	quadratic	y = 0.05x^2^ + 0.71x + 0.1	0.88	<0.001

Data in the same row, not sharing the same superscript letter, are significantly different (*p* < 0.05). SFA: saturated fatty acid; MUFA: mono-unsaturated fatty acid; n-6 PUFA: n-6 poly-unsaturated fatty acid; n-3 PUFA: n-3 poly-unsaturated fatty acid; “/” indicates that no statistically significant model (*p* < 0.05) was obtained for this parameter.

**Table 7:** Effects of different levels of dietary DHA on the fatty acid compositions in the liver of juvenile tiger puffer (*Takifugu rubripes*) (% total fatty acids).

Fatty Acid	DHA0	DHA1	DHA2	DHA3	DHA4	DHA5	Regression			
							Model	Equation	*R* ^2^	*p*
14:0	1.25 ± 0.08	1.17 ± 0.08	1.15 ± 0.18	1.11 ± 0.06	1.18 ± 0.17	1.17 ± 0.12	/			
16:0	14.18 ± 0.16	14.32 ± 0.22	14.16 ± 0.24	14.48 ± 0.27	14.26 ± 0.42	14.00 ± 0.44	/			
18:0	4.36 ± 0.19 ^a^	4.67 ± 0.15 ^ab^	5.03 ± 0.17 ^abc^	5.32 ± 0.20 ^bc^	5.26 ± 0.23 ^bc^	5.61 ± 0.32 ^c^	/			
20:0	0.22 ± 0.01 ^c^	0.22 ± 0.01 ^bc^	0.21 ± 0.01 ^abc^	0.18 ± 0.01 ^a^	0.19 ± 0.01 ^ab^	0.19 ± 0.04 ^ab^	cubic	y = 4.44 × 10^−3^x^3^ − 6.11E − 03x^2^ + 0.02x + 0.21	0.61	<0.001
SFA	20.01 ± 0.33	20.37 ± 0.31	20.54 ± 0.31	21.08 ± 0.37	20.88 ± 0.60	20.92 ± 0.73	quadratic	y = 0.04x^2^ − 0.08x + 20.16	0.16	0.008
16:1n-7	0.14 ± 0.01 ^ab^	0.13 ± 0.00 ^a^	0.15 ± 0.01 ^ab^	0.14 ± 0.02 ^ab^	0.14 ± 0.01 ^ab^	0.16 ± 0.02 ^b^	/			
18:1n-9	31.78 ± 0.64 ^f^	27.37 ± 1.09 ^e^	24.11 ± 0.61 ^d^	20.96 ± 0.94 ^c^	16.49 ± 1.40 ^b^	13.45 ± 2.50 ^a^	cubic	y = −0.12x^3^ + 0.97x^2^ − 4.69x + 35.5	0.91	<0.001
20:1n-9	1.50 ± 0.15^c^	1.34 ± 0.14^c^	1.32 ± 0.10^c^	1.05 ± 0.08^b^	1.04 ± 0.15^b^	0.85 ± 0.11 ^a^	cubic	y = 3.71 × 10^−3^x^3^ − 0.05x^2^ + 0.09x + 1.44	0.61	<0.001
22:1n-9	0.31 ± 0.02 ^d^	0.23 ± 0.03 ^c^	0.23 ± 0.02 ^c^	0.18 ± 0.01 ^b^	0.18 ± 0.03 ^b^	0.14 ± 0.02 ^a^	cubic	y = −1.41 × 10^−3^x^3^ + 0.02x^2^ − 0.08x + 0.37	0.77	<0.001
MUFA	33.73 ± 0.23 ^f^	29.07 ± 0.39 ^e^	25.80 ± 0.22 ^d^	22.33 ± 0.40 ^c^	17.84 ± 0.52 ^b^	13.10 ± 0.51 ^a^	cubic	y = −0.12x^3^ + 0.95x^2^ − 4.71x + 37.46	0.92	<0.001
18:2n-6	15.67 ± 0.37 ^f^	13.62 ± 0.19 ^e^	12.36 ± 0.30 ^d^	11.08 ± 0.26 ^c^	9.22 ± 0.52 ^b^	8.32 ± 0.77 ^a^	cubic	y = −0.05x^3^ + 0.51x^2^ − 2.45x + 17.71	0.93	<0.001
20:2n-6	0.59 ± 0.06 ^b^	0.54 ± 0.05 ^ab^	0.57 ± 0.04 ^ab^	0.52 ± 0.04 ^ab^	0.53 ± 0.06 ^ab^	0.49 ± 0.06 ^a^	/			
20:4n-6	1.29 ± 0.09 ^a^	1.49 ± 0.12 ^b^	1.37 ± 0.10 ^ab^	1.48 ± 0.09 ^b^	1.44 ± 0.15 ^ab^	1.57 ± 0.14 ^b^	/			
n-6PUFA	17.54 ± 0.43 ^f^	15.65 ± 0.16 ^e^	14.30 ± 0.33 ^d^	13.08 ± 0.26 ^c^	11.19 ± 0.60 ^b^	10.39 ± 0.76 ^a^	cubic	y = −0.05x^3^ + 0.44x^2^ − 2.19x + 19.38	0.92	<0.001
18:3n-3	2.97 ± 0.10 ^f^	2.58 ± 0.02 ^e^	2.10 ± 0.05 ^d^	1.62 ± 0.03 ^c^	1.20 ± 0.06 ^b^	0.70 ± 0.05 ^a^	quadratic	y = −0.03x^2^ − 0.1x + 3.11	0.94	<0.001
20:5n-3	5.62 ± 0.12	5.60 ± 0.07	5.75 ± 0.09	5.40 ± 0.13	5.46 ± 0.15	5.49 ± 0.12	/			
22:5n-3	3.25 ± 0.16 ^a^	3.90 ± 0.09 ^b^	4.23 ± 0.13 ^b^	4.87 ± 0.12 ^c^	5.15 ± 0.19 ^c^	5.42 ± 0.29 ^c^	quadratic	y = 0.02x^2^ + 0.22x + 3.03	0.79	<0.001
22:6n-3	4.25 ± 0.11 ^a^	10.30 ± 0.17 ^b^	15.29 ± 0.19 ^c^	20.23 ± 0.40 ^d^	26.36 ± 0.79 ^e^	32.57 ± 1.18 ^f^	quadratic	y = 0.37x^2^ + 1.5x + 2.67	0.93	<0.001
n-3PUFA	12.84 ± 0.19 ^a^	18.48 ± 0.18 ^b^	23.14 ± 0.20 ^c^	27.25 ± 0.50 ^d^	33.02 ± 0.82 ^e^	38.76 ± 1.22 ^f^	cubic	y = 0.1x^3^ − 0.73x^2^ + 4.74x + 8.77	0.92	<0.001
DHA/EPA	0.76 ± 0.01 ^a^	1.84 ± 0.04 ^b^	2.67 ± 0.06 ^b^	3.75 ± 0.06 ^c^	4.85 ± 0.19 ^d^	5.93 ± 0.19 ^e^	quadratic	y = 0.05*x*^2^ + 0.75*x* + 0.03	0.90	<0.001

Data in the same row, not sharing the same superscript letter, are significantly different (*p* < 0.05). SFA: saturated fatty acid; MUFA: mono-unsaturated fatty acid; n-6 PUFA: n-6 poly-unsaturated fatty acid; n-3 PUFA: n-3 poly-unsaturated fatty acid; “/” indicates that no statistically significant model (*p* < 0.05) was obtained for this parameter.

**Table 8:** Effects of different levels of dietary DHA on the fatty acid compositions in the muscle of juvenile tiger puffer (*Takifugu rubripes*) (% total fatty acids).

Fatty Acid	DHA0	DHA1	DHA2	DHA3	DHA4	DHA5	Regression			
							Model	Equation	*R* ^2^	*p*
14:0	0.31 ± 0.07	0.28 ± 0.02	0.32 ± 0.05	0.33 ± 0.06	0.35 ± 0.07	0.43 ± 0.11	quadratic	y = 8.28 × 10^−3^x^2^ − 0.03x + 0.33	0.36	0.038
16:0	19.44 ± 0.44 ^a^	20.26 ± 0.61 ^ab^	20.98 ± 0.92 ^ab^	21.70 ± 0.89 ^ab^	22.40 ± 1.43 ^b^	25.95 ± 1.12 ^c^	cubic	y = 0.14x^3^ − 1.18x^2^ + 3.73x + 16.67	0.87	<0.001
18:0	7.37 ± 0.37 ^a^	7.71 ± 0.44 ^a^	7.57 ± 0.54 ^a^	7.45 ± 0.30 ^a^	7.67 ± 0.53 ^a^	8.90 ± 0.11 ^b^	cubic	y = 0.08x^3^ − 0.71x^2^ + 1.93x + 6.07	0.70	<0.001
20:0	0.22 ± 0.03	0.22 ± 0.03	0.22 ± 0.03	0.21 ± 0.06	0.20 ± 0.05	0.26 ± 0.03	/			
SFA	27.28 ± 0.85 ^a^	28.46 ± 1.01 ^a^	29.09 ± 1.38 ^a^	29.69 ± 1.29 ^a^	30.62 ± 2.04 ^a^	35.45 ± 1.07 ^b^	cubic	y = 0.22x^3^ − 1.93x^2^ + 5.8x + 23.09	0.84	<0.001
16:1n-7	0.80 ± 0.09	0.80 ± 0.03	0.80 ± 0.01	0.78 ± 0.03	0.79 ± 0.01	0.84 ± 0.05	/			
18:1n-9	19.11 ± 0.58 ^d^	17.60 ± 1.20 ^d^	15.14 ± 0.65 ^c^	12.07 ± 1.21 ^b^	11.79 ± 0.66 ^b^	9.31 ± 0.66 ^a^	cubic	y = 2.95 × 10^−3^x^3^ − 0.03x^2^ + 0.06x + 0.76	0.94	<0.001
20:1n-9	0.80 ± 0.07 ^c^	0.66 ± 0.06 ^bc^	0.63 ± 0.06 ^b^	0.54 ± 0.02 ^ab^	0.53 ± 0.05 ^ab^	0.44 ± 0.09 ^a^	quadratic	y = 0.07x^2^ − 2.46x + 21.76	0.82	<0.001
MUFA	20.74 ± 0.71 ^d^	19.06 ± 1.19 ^d^	16.60 ± 0.62 ^c^	13.45 ± 1.29 ^b^	13.11 ± 0.66 ^b^	10.60 ± 0.62 ^a^	cubic	y = −4.51 × 10^−3^x^3^ + 0.05x^2^ − 0.25x + 1	0.94	< 0.01
18:2n-6	11.85 ± 0.27 ^e^	9.81 ± 0.76 ^d^	8.15 ± 0.22 ^c^	7.01 ± 0.09 ^b^	6.39 ± 0.58 ^b^	5.29 ± 0.33 ^a^	cubic	y = 0.03x^3^ − 0.27x^2^ − 1.54x + 22.65	0.97	<0.001
20:2n-6	0.53 ± 0.10	0.51 ± 0.04	0.51 ± 0.07	0.46 ± 0.02	0.46 ± 0.01	0.43 ± 0.05	cubic	y = −0.04x^3^ + 0.58x^2^ − 3.64x + 14.99	0.33	0.047
20:4n-6	5.95 ± 0.18 ^c^	5.19 ± 0.28 ^b^	4.95 ± 0.11 ^ab^	4.81 ± 0.46 ^ab^	4.51 ± 0.06 ^ab^	4.27 ± 0.31 ^a^	quadratic	y = −9.94 × 10^−4^x^2^ − 0.01x + 0.54	0.85	<0.001
n-6PUFA	18.33 ± 0.16 ^e^	15.50 ± 0.64 ^d^	13.62 ± 0.40 ^c^	12.28 ± 0.50 ^b^	11.37 ± 0.56 ^b^	9.99 ± 0.60 ^a^	cubic	y = −0.03x^3^ + 0.35x^2^ − 1.52x + 7.14	0.98	<0.001
18:3n-3	0.89 ± 0.02 ^b^	0.76 ± 0.07 ^b^	0.67 ± 0.08 ^b^	0.55 ± 0.18 ^ab^	0.51 ± 0.23 ^ab^	0.25 ± 0.09 ^a^	cubic	y = −0.07x^3^ + 0.92x^2^ − 5.15x + 22.64	0.78	<0.001
20:5n-3	7.06 ± 0.35 ^f^	5.24 ± 0.19 ^e^	5.02 ± 0.57 ^d^	4.20 ± 0.22 ^c^	4.19 ± 0.44 ^b^	3.41 ± 0.15 ^a^	cubic	y = −9.52 × 10^−3^x^3^ + 0.09x^2^ − 0.36x + 1.17	0.92	<0.001
22:5n-3	3.46 ± 0.05 ^d^	2.69 ± 0.19 ^c^	2.45 ± 0.05 ^bc^	2.12 ± 0.12 ^ab^	2.13 ± 0.11 ^ab^	1.93 ± 0.06 ^a^	cubic	*y* = −0.02*x*^3^ + 0.31*x*^2^ − 1.48*x* + 4.64	0.91	<0.001
22:6n-3	13.37 ± 0.19 ^a^	20.19 ± 0.72 ^b^	24.41 ± 0.28 ^b^	26.53 ± 1.66 ^cd^	29.72 ± 0.52 ^de^	30.78 ± 0.80 ^e^	cubic	y = −0.07x^3^ + 0.86x^2^ − 3.65x + 9.88	0.96	<0.001
n-3PUFA	21.32 ± 0.27 ^a^	26.19 ± 0.70 ^b^	30.10 ± 0.56 ^c^	31.28 ± 1.64 ^cd^	34.42 ± 0.27 ^d^	34.35 ± 0.75 ^d^	quadratic	y = −0.5x^2^ + 6.07x + 15.88	0.93	<0.001
DHA/EPA	1.90 ± 0.10 ^a^	3.86 ± 0.27 ^b^	4.90 ± 0.54 ^c^	6.36 ± 1.03 ^d^	7.15 ± 0.89 ^e^	9.06 ± 0.78 ^f^	cubic	y = 0.06x^3^ − 0.7x^2^ + 3.56x − 1.02	0.94	<0.001

Data in the same row, not sharing the same superscript letter, are significantly different (*p* < 0.05). SFA: saturated fatty acid; MUFA: mono-unsaturated fatty acid; n-6 PUFA: n-6 poly-unsaturated fatty acid; n-3 PUFA: n-3 poly-unsaturated fatty acid; “/” indicates that no statistically significant model (*p* < 0.05) was obtained for this parameter.

## Data Availability

The original contributions presented in this study are included in the article. Further inquiries can be directed to the corresponding authors.

## Abbreviations

Abbreviation	Full name
Non-gene name	
LC-PUFA	long-chain polyunsaturated fatty acids
EPA	eicosapentaenoic acid
DHA	docosahexaenoic acid
ARA	arachidonic acid
FO	fish oil
SFA	saturated fatty acid
MUFA	monounsaturated fatty acid
RT-qPCR	real-time quantitative polymerase chain reaction
WG	weight gain
SGR	specific growth rate
VSI	viscerosomatic index
HSI	hepatosomatic index
TFA	total fatty acids
TG	triglyceride
T-CHO	total cholesterol
HDL-C	high-density lipoprotein cholesterol
LDL-C	low-density lipoprotein choles-terol
TBA	total bile acid
MDA	malondialdehyde
Gene name	
*myod*	myogenic differentiation antigen
*myog*	myogenin
*myf6*	myogenic factor 6
*myf5*	myogenic factor 5
*bax*	*bcl-2*-associated x
*bcl-2*	b-cell lymphoma-2
*acta2*	actin alpha 2
*il-1β*	interleukin—1β
*il-8*	interleukin 8
*tnf-α*	tumor necrosis factor alpha
*ifn-γ*	interferon gamma
*jam-a*	junctional adhesion molecule a
*mlck*	myosin light chain kinase
*rpl19*	ribosomal protein l19
*rpl13*	ribosomal protein l13
*keap1*	Kelch-like ECH-associated protein 1
*nrf2*	nuclear factor erythroid 2
*col1a2*	collagen type I alpha 2 chain
